# Endoglin is a conserved regulator of vasculogenesis in zebrafish – implications for hereditary haemorrhagic telangiectasia

**DOI:** 10.1042/BSR20182320

**Published:** 2019-05-21

**Authors:** Ding Zhang, Fang Zhou, Xiuli Zhao, Bao Liu, Jingyu Chen, Jun Yang

**Affiliations:** 1 Department of Cell Biology, State Key Laboratory of Medical Molecular Biology, Institute of Basic Medical Sciences, Chinese Academy of Medical Sciences and Peking Union Medical College, Beijing 100005, China; 2 Department of Medical Genetics, Institute of Basic Medical Sciences, Chinese Academy of Medical Sciences and School of Basic Medicine, Peking Union Medical College, Beijing 100005, China; 3 Department of Vascular Surgery, Peking Union College Hospital, Chinese Academy of Medical Science and Peking Union Medical College, Beijing 100005, China; 4 Wuxi Lung Transplant Center, Wuxi People’s Hospital affiliated to Nanjing Medical University, Wuxi 214023, China

**Keywords:** endoglin, endothelial cells, vasculogenesis, zebrafish

## Abstract

Hereditary haemorrhagic telangiectasia (HHT) is a progressive vascular disease with high mortality and prevalence. There is no effective treatment of HHT due to the lack of comprehensive knowledge of its underlying pathological mechanisms. The majority of HHT1 patients carry *endoglin* (*ENG*) mutations. Here, we used *Danio rerio* (zebrafish) as an *in vivo* model to investigate the effects of endoglin knockdown on vascular development. According to phylogenetic analyses and amino acid sequence similarity analyses, we confirmed that endoglin is conserved in vertebrates and descended from a single common ancestor. Endoglin is highly expressed in the vasculature beginning at the segmentation period in zebrafish. Upon endoglin knockdown by morpholinos, we observed disruption in the intersegmental vessels (ISVs) and decreased expression of several vascular markers. RNA sequencing (RNA-Seq) results implied that the *BMP-binding endothelial regulator* (*bmper*) is a gene affected by endoglin knockdown. Rescue experiments demonstrated that overexpression of bmper significantly increased the number of endothelial cells (ECs) and reduced the defects at ISVs in zebrafish. Moreover, there was enhanced tube formation in *ENG* mutant ECs derived from a HHT patient after human recombinant BMPER (hrBMPER) stimulation. Taken together, our results suggest that *bmper*, a potential downstream gene of *ENG*, could be targeted to improve vascular integrity in HHT.

## Introduction

Hereditary haemorrhagic telangiectasia (HHT), known as Rendu–Osler–Weber syndrome or HHT, is an autosomal dominant inherited vascular disease, with an estimated prevalence of 1:5000 [1]. HHT is characterised by telangiectasia at the junction of skin and mucous membranes, commonly in the tongue, lips, fingertips, ears and conjunctiva and is often accompanied by severe, recurrent epitasis and gastrointestinal haemorrhage. Due to abnormal vascular development, patients with HHT tend to form abundant vascular networks between the veins and arteries, including telangiectasia, arteriovenous malformations (AVMs) and arteriovenous fistula (AVF). Conventional therapies, such as transcatheter embolisation, anti-fibrin and bevacizumab, have been used to relieve the symptoms of HHT, but none of them is formally approved for the treatment of HHT by regulatory offices. Thus, discovering the specific drug target would be fundamental to find a novel strategy for the treatment of HHT [2].

Genetic studies have mapped HHT at the chromosome level and divided HHT into two types: type I, with an *endoglin* (*ENG*) mutation located at 9q33-34.1, also known as TGF-β receptor III [3,4]; and type II, with an *Activin receptor-like kinase I* (*ALK1*) mutation located at 9q33-34.1. Previous work has indicated that endoglin is highly expressed in vascular endothelial cells (ECs) [5,6]. Endoglin deficiency has been shown to induce vascular injury *in vivo* and inhibit capillary tube formation *in vitro* [7]. Some articles have indicated that endoglin is highly involved in maintaining endothelial function, vascular homoeostasis and angiogenesis *in vivo* [8]. Although Sugden et al. demonstrated that endoglin controls the blood vessel diameter by changing the EC shape [9], the mechanism of endoglin regulation of vascular development in early embryogenesis remains unclear.

In our study, *Danio rerio* (zebrafish) was chosen as an *in vivo* model to study vascular development due to three aspects: (i) the high reproduction rates and easy embryo operation [10,11]; (ii) the ability to observe the formation of vasculature at different developmental stages through *Tg(fli1a:EGFP)^y1^* transgene zebrafish line [12–14] and (iii) the ability of morpholino, an accepted knockdown tool in zebrafish, to knockdown gene expression by blocking translation or preventing proper pre-mRNA splicing [15].

We analysed the structural and evolutionary conservation of endoglin among vertebrates and examined the effects of endoglin knockdown on zebrafish embryogenesis. By employing zebrafish together with ECs derived from induced pluripotent stem cells (iPSCs) of an HHT patient, we first identified BMPER as a downstream gene of endoglin. Our results suggest that the loss of endoglin affected vasculogenesis in zebrafish, and BMPER could be a potential therapeutic target of HHT.

## Methods

### Ethics statement

The experimental protocols were in accordance with the principles of the China Zebrafish Resource Center and approved by the Research Ethics Committee of Peking Union Medical College. All animal procedures were carried out in the Zebrafish Laboratory of State Key Laboratory of Medical Molecular Biology, Institute of Basic Medical Sciences, Chinese Academy of Medical Sciences and Peking Union Medical College. Con ECs and *ENG* mutant ECs were differentiated from iPSCs of a healthy donor and an HHT patient (carried *ENG* mutation) provided by Wuxi People’s Hospital and Peking Union Medical College Hospital with approval from the college research ethics committee respectively.

### Zebrafish lines and husbandry

All adult zebrafish were raised in a recirculating aquaculture system and fed with *Artemia nauplii* at 26–28°C. A 14 h light and 10 h dark cycle was used as it is an optimal biorhythm for zebrafish. The zebrafish lines were AB (wild type), *Tg(flk:GFP)* and *Tg(fli1a:EGFP)^y1^* [16].

### Embryo treatment

Embryos were incubated in acidic seawater (pH 5.0) at 28.5°C [17]. The embryos were developed to certain stages, including 1 cell, 2 cell, 128 cell, sphere, 75% epiboly, 12, 18, 24 or 72 hpf stage, determined according to standards set by Howe et al. [18]. The embryos at the different stages were divided into two groups: group 1 for total RNA extraction and group 2 for *in situ* hybridisation. Group 2 was fixed in 4% paraformaldehyde (PFA) for 24–48 h and washed with PBS three times (5 min per time, RT). For long-term storage, embryos were dehydrated by a gradient methanol solution and stored in absolute methanol at −20°C.

### Morpholinos injection and mRNA synthesis

Morpholinos were designed to block translation by targeting the AUG initiation codon (Gene Tools, Philomath, U.S.A.). The morpholinos used are listed below:

1. Endoglin-MOs sequence: 5′-GATGAACTCAACACTCGTGTCTGAT-3′.
2. 5-Mispair control MOs sequence: 5′-AAACAgAcCAcATcCTCTTCATcTC-3′.

Off-target effects and specificity of endoglin-MOs were addressed in a commonly used approach, a rescue experiment. Full-length human endoglin mRNA was co-injected with endoglin-MOs to rescue the zebrafish vascular phenotype.

Capped and polyadenylated full-length mRNA was generated according to Timme-Laragy et al. [15], including construction of pcDNA plasmids containing human endoglin [19], zebrafish bmper, zebrafish alk1, zebrafish bmp9 and mCherry (control), linearisation of the plasmids using *NheI* (New England Biolabs, U.S.A.), synthesis of the mRNA by mMESSAGE mMACHINE T7 Transcription Kit (Thermo Fisher, U.S.A.).

The microinjection was carried out according to Satou et al. [20]. In brief, 1 cell stage embryos were used as they are the optimal embryos for injection of MOs and mRNA using the Femto Jet injection system (Eppendorf) under a controllable nitrogen pressure. All embryos were injected with 2 ng morpholinos and 500 pg mRNA (rescue experiment). Injected embryos were cultured in acidic sea water at 27°C.

### Cell dissociation and FACS analysis

In each group, 40 *Tg(fli1a:EGFP)^y1^* or *Tg(flk:GFP)* embryos (24 and 48 hpf) were digested with trypsin/EDTA (0.05%) for 10–15 min and dissociated to a single-cell suspension at RT. Dissociation was stopped by the addition of 10 μl CaCl_2_ (0.1 mM). Then, the cells were filtered and isolated through a 100 μm cell strainer and resuspended in 100 μl PBS [21].

The single-cell suspension of zebrafish embryos was analysed using the Accuri C6 Flow Cytometer (BD sciences, U.S.A.). GFP^+^ cells were detected using the FL1 channel.

FACS was completed by BD FACSAria™ III (BD Biosciences, U.S.A.), and 3 × 10^5^ GFP^+^ cells from different groups were collected in a 1.5 ml RNase-free centrifuge tube for RNA extraction. The details of FACS were described by Sun et al. [22] and Nguyen-Chi et al. [23].

### Zebrafish total RNA and tissue RNA extraction

First, adult zebrafish was anaesthetised by placement into a 2-phenoxyethanol solution (1:1000) for 2–3 min and then dissected for tissue dissection, as described by Gupta and Mullins [24]. The separated tissues were quickly placed into TRIzol® Reagent to avoid RNA degradation. The following RNA extraction experiments were performed under the standard protocols of Total RNA Kit I (Omega Bio-Tek, U.S.A.).

The RNA extraction procedure for the different embryo stages (group 1) was the same as described above.

### cDNA synthesis and quantitative real-time-PCR (qRT-PCR)

RNA was dissolved in 30 µl of DEPC water. Then, 1 µg of RNA solution was reverse transcribed to cDNA using an RT reagent kit (Takara, Japan). qPCR was performed with the TransStart TipGreen qPCR SuperMix (TransGen Biotechnology, China) at the CFX Connect Real-time System (Bio-Rad, U.S.A.). The qPCR cycling programme was as follows: (1) initial denaturation at 95°C for 1 min; (2) 39 cycles of denaturation at 95°C for 15 s; (3) annealing plus extension at 60°C for 30 s.

All of the gene-specific primers for qPCR were designed by BLAST (NCBI).

### Probe synthesis

Digoxigenin-labelled antisense probes: The target gene fragments were amplified by RT-PCR (Takara Prime STAR® Max DNA Polymerase kit, Japan) and cloned into pEASY-T3 (TransGen Biotechnology, China). The reconstructed expression vector was cut by a specific restriction endonuclease to be linearised and transcribed into the RNA probe using the MAXIscript™ SP6/T7 Transcription Kit (Thermo Fisher, U.S.A.).

### Whole-mount *in situ* hybridisation

Chromogenic whole-mount *in situ* hybridisation (WISH) was performed using standards set by Jakt et al. [25], including the use of proteinase K to disrupt embryos, pre-hybridisation, hybridisation, antibody incubation and the chromogenic reaction.

All zebrafish embryos were fixed in 4% PFA (16–32 h) prior to *in situ* hybridisation.

### RNA sequencing

More than 30 embryos at 24 hpf were collected in 1 ml TRIzol® reagent, and these samples were sent to Novogene (Tianjin, China) for further RNA sequencing (RNA-Seq) analysis, including RNA quality detection, cDNA library generation, sequencing and sequencing alignment.

### Cell lines and cell culture

The method for the differentiation of iPSCs into ECs was described by Orlova et al. [26]. ECs were isolated by CD31 Dynabeads (Miltenyi Biotech) and cultured in EC-SFM (FULL) medium (Gibco, U.S.A.) supplemented with 30 ng/ml VEGF165 (R&D, U.S.A.), 30 ng/ml bFGF (R&D, U.S.A.) and 1% FBS (Gibco, U.S.A.).

### Cell collection and Western blot analysis

First, *ENG* mutant and Con ECs were serum-starved overnight. Then, *Eng* mutant and Con ECs were treated with 0.1% DMSO, 20 ng/ml BMPER (R&D, U.S.A.) [27,28] and 10 ng/ml BMP9 (R&D, U.S.A.) [29] for 24 h. Cell collection and Western blot were performed as described by Meinert et al. [30]. The antibodies used for Western blotting of ID1 and GAPDH were sc-13n3104 (Santa Cruz, U.S.A.) and ab181602 (Abcam, U.S.A.), respectively. Chemiluminescence stained bands were visualised by the Tanon-5200 Image Analyzer (Tanon, China).

### Tube formation of endothelial cells

The tube formation assay was performed as described by Heinke et al. [27]. Briefly, 200 µl Matrigel (BD Biosciences, U.S.A.) was pipetted into a 96-well culture plate at 37°C for 1 h. After dissociation into single cells by Accutase, *Eng* mutant cells (1 × 10^4^ cells/well) were seeded onto Matrigel. Tube formation was observed for 3–5 h after seeding and photographed with a light microscope (Nikon E100, Japan). The number of tube junctions and tube branches were assessed by ImageJ.

### Statistical analysis

All statistical results were presented as the mean ± SEM using Student’s *t-*test or two-way ANOVA with GraphPadf cfc Prism 6 software. Figure legends show the number of biological repeats for each experiment (*n*). A value of *P* was considered statistically significant (^*^P<0.05, ^**^P<0.01, ^***^P<0.001).

## Results

### Endoglin is structurally and evolutionarily conserved among vertebrates

**Figure 1:**
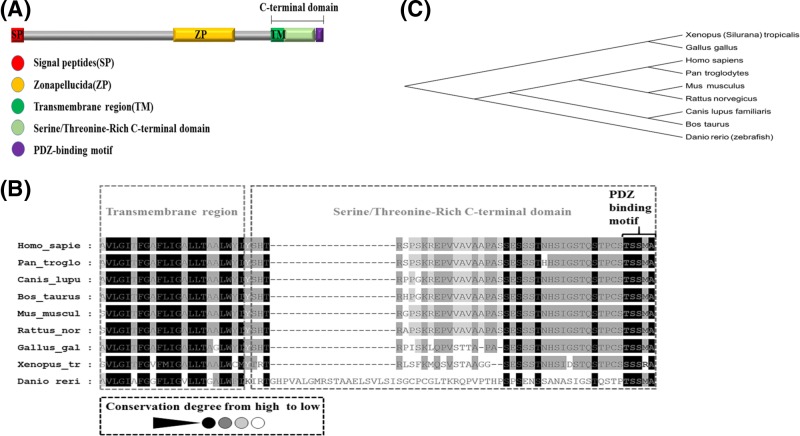
Endoglin amino acid sequence and phylogenetic analysis (**A**) Schematic structure of the zebrafish endoglin protein, including the signal peptides (red), zona pellucida (yellow), transmembrane region (dark green), serine/threonine-rich C-terminal domain (light green) and PDZ-binding motif (purple). (**B**) Amino acid sequence analysis of the endoglin C-terminal region using the software Gendoc. The colour of the background reflects the degree of conservation: black is highly conserved, grey is partially conserved and white is the lowest conserved region. The transmembrane region is the most conversed among the orthologues. (**C**) Endoglin phylogenetic analysis was conducted using the software MEGA6.0. Endoglin is evolutionarily conserved in Xenopus, chickens, humans, chimpanzees, mice, rats, canines, cattle and zebrafish. All endoglin orthologues originated from a common ancestor.

As a member of the TGF-β family, the endoglin protein has three elementary domains: the signal peptide (SP), zona pellucida (ZP) and C-terminal domain (Figure 1A). By amino acid sequence alignment of endoglin between zebrafish and vertebrate orthologues, we found that endoglin is partly conserved. The sequence of the transmembrane region (TM) is similar in different vertebrates (Figure 1B). Phylogenetic analysis provided evidence that vertebrate endoglin originates from a common ancestor. Zebrafish endoglin belongs to a subfamily that is distinct from that in mammals (Figure 1C). These results indicate that endoglin is a conserved protein among vertebrates, and the zebrafish model could be used to study the *eng* mutation that causes HHT.

### Endoglin is highly expressed in the vasculature beginning at the segmentation period

**Figure 2:**
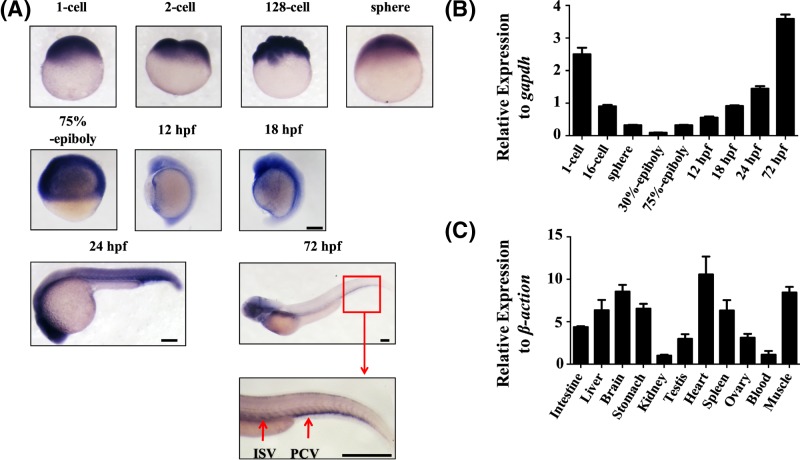
Temporal-spatial expression of endoglin (**A**) Embryonic zebrafish endoglin expression by WISH (*n*>30 for each group). Embryo stages, as labelled, are shown in lateral views from the 1 cell to 72 hpf stage. Blue staining shows a positive signal for endoglin expression and the red box indicates high expression of endoglin in the tail region. Scale bars are 200 μm. (**B**) qPCR analysis of endoglin expression in different stages of zebrafish embryos from the 1-cell to 72 hpf stage. *Gapdh* was used as an internal control (mean ± SD; the experiments were repeated three times). (**C**) The mRNA level of endoglin in various tissues of the adult zebrafish, including the intestine, liver, brain, stomach, kidney, testis, heart, spleen, ovary, blood and muscle. *β-Actin* was used as an internal control (mean ± SD; the experiments were repeated three times).

For *in situ* hybridisation and qPCR, we collected samples from the following stages: zygote, cleavage period, blastula stage, gastrula period, segmentation period and 24-h stage. WISH of zebrafish embryos showed a unique endoglin expression pattern throughout early embryogenesis. In the zygote, endoglin was highly expressed in the cell pole. In the cleavage period (2-64 cell stage), endoglin was evenly distributed in the blastomeres. In the blastula stage (128-cell to 30% epiboly), endoglin expression in the 128-cell to sphere stage remained even but was distributed unevenly in the 30% epiboly stage. In the gastrula period (50% epiboly to bud stages), endoglin was highly expressed in the regions where haematopoiesis and vasculogenesis originated [10]. Beginning at the segmentation period (12–24 hpf), endoglin was mainly expressed in the vasculature, including the posterior cardinal vein (PCV) and intersegmental vessels (ISVs) (Figure 2A). The qPCR results indicated that endoglin expression was high in the zygote, decreased during the cleavage period, and increased in the gastrula period and segmentation period (Figure 2B).

Spatial tissue expression of endoglin was analysed by qPCR. We separated 11 different tissues from adult zebrafish and extracted RNA to synthesise cDNA, including RNA from the intestine, liver, brain, stomach, kidney, testis, heart, spleen, ovary, blood and muscle. Endoglin was highly expressed in the heart and brain but expressed at low levels in the kidney and blood (Figure 2C).

### Endoglin knockdown affects vascular development

**Figure 3:**
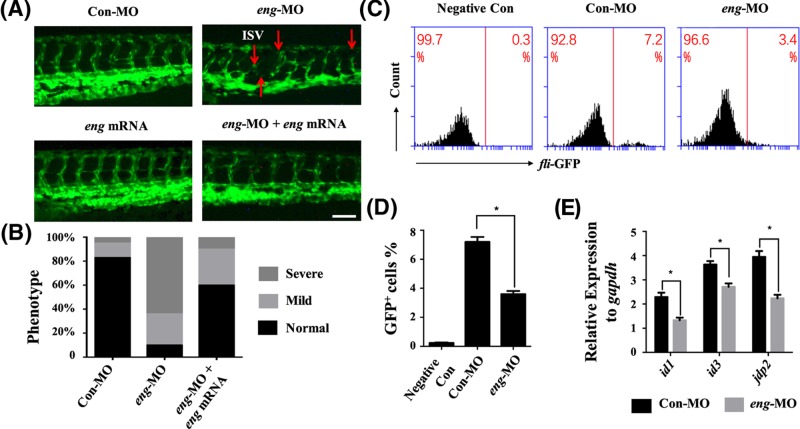
Endoglin knockdown affected the development of the vasculature (**A**) 72 hpf *Tg(fli1a:EGFP)^y1^* transgene zebrafish embryos were selected to demonstrate the phenotypic change in the Con-MO, *eng*-MO, *eng* mRNA and *eng*-MO + *eng* mRNA group. (All *Tg(fli1a:EGFP)^y1^* zebrafish embryos were injected with 2 ng morpholinos and 500 pg mRNA. For example, Con-MO group: 2 ng 5-mispair control MO and 500 pg mCherry mRNA). The red arrows point to the disrupted structure of ISVs. Scale bars are 100 μm. (**B**) Quantification of phenotypic defects in the Con-MO, *eng*-MO and *eng*-MO + *eng* mRNA group (Normal: most vascular and intersegmental blood vessels do not have obvious defects; Mild: most vascular vessels do not have obvious defects, but some intersegmental blood vessels are twisted and defective; Severe: most vascular and intersegmental blood vessels are disrupted, and some intersegmental blood vessels have disappeared). (**C**) FACS analysis of *fli*-GFP^+^ cells in the 24 hpf Negative Con, Con-MO and *eng*-MO groups (Negative Con: AB zebrafish injected with 5-mispair control MOs; Con-MO: *Tg(fli1a:EGFP)^y1^* zebrafish injected with 5-mispair control MOs; *eng*-MO: *Tg(fli1a:EGFP)^y1^* zebrafish injected with endoglin MOs). (**D**) Quantification of *fli*-GFP^+^ cell number from FACS analysis (mean ± SD; the experiments were repeated three times). (**E**) qPCR analysis of endoglin downstream gene expression in 24 hpf zebrafish embryos, including *id1, id3* and *jdp2. Gapdh* was used as an internal control (mean ± SD; the experiments were repeated three times). A value of *P* was considered statistically significant (^*^P<0.05) for D and E.

Here, the *Tg(fli1a:EGFP)^y1^* transgenic line was used to show the vasculature [16]. The blood vessels developed abnormally with some ISVs breaking in the 72 hpf endoglin-morpholino injected embryos (*eng*-MO group). Overexpression of endoglin resulted in the recovery of defective ISVs compared with *eng*-MO group (Figure 3A,B). FACS analysis showed that the ratio of *fli*-GFP^+^ cells was decreased to 3.4% in the 72 hpf *eng*-MO group compared with 7.2% in the 72 hpf Con-MO group (Figure 3C,D). Considering *fli* is an endothelial and haematopoietic marker in zebrafish [31,32], we also conducted the knockdown experiments on *flk*-GFP^+^ line and got the similar results (Figure S1A,B). Moreover, the expression of endoglin’s downstream genes (*id1, id3, jdp2*) was decreased in the *eng*-MO group (Figure 3E) [33,34]*.* These results suggested that the defects of ISVs in the *eng*-MO group were caused by endoglin knockdown.

### Endoglin knockdown causes reduced expression of endothelial markers

**Figure 4:**
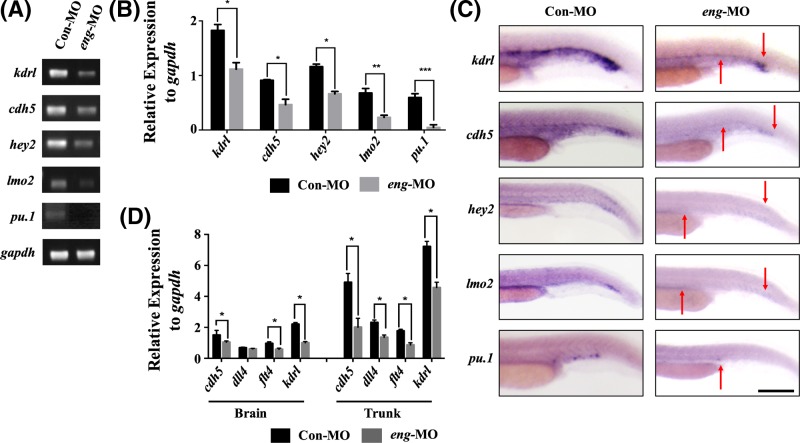
Endoglin knockdown decreased the expression of endothelial markers (**A**) RT-PCR for endothelial markers in the Con-MO and *eng*-MO group, including *kdrl, cdh5, hey2, lmo2* and *pu.1* (A). *Gapdh* was used as an internal control. (**B**) Quantification of mRNA expression in (A) with grey scanning analysis, conducted with ImageJ. (**C**) WISH for endothelial marker expression in the Con-MO and *eng*-MO group among 24 hpf embryos (*n*>30 for each group). The red arrow indicates the region where the endothelial markers were significantly decreased. Scale bars are 200 μm. (**D**) qPCR for *cdh5, dll4, flt4* and *kdrl* expression in the brain and trunk of the Con-MO and *eng*-MO group. *Gapdh* was used as an internal control (error bars, SEM; *n*=3 biological replicates). A value of *P* was considered statistically significant (^*^P<0.05, ^**^P<0.01, ^***^P<0.001) for B and D.

We further examined the expression of endothelial markers in 24 hpf embryos by RT-PCR and *in situ* hybridisation. The expression levels of the endothelial markers, including *kdrl, cdh5, hey2, lmo2* and *pu.1* [12,35], were significantly decreased after knocking down endoglin (Figure 4A–C). According to qPCR results, the expression of four endothelial markers, including *kdrl* (expressed in all vasculature), *dll4* (mainly expressed in the dorsal aorta and ISVs), *flt4* (mainly expressed in ISVs) and *cdh5* (mainly expressed in the dorsal aorta, ISVs, PCV), was more diminished the trunk than in the brain (Figure 4D). These results suggested that endoglin knockdown affected endothelium formation in zebrafish.

### BMPER is involved in endoglin-regulated vasculogenesis

**Figure 5:**
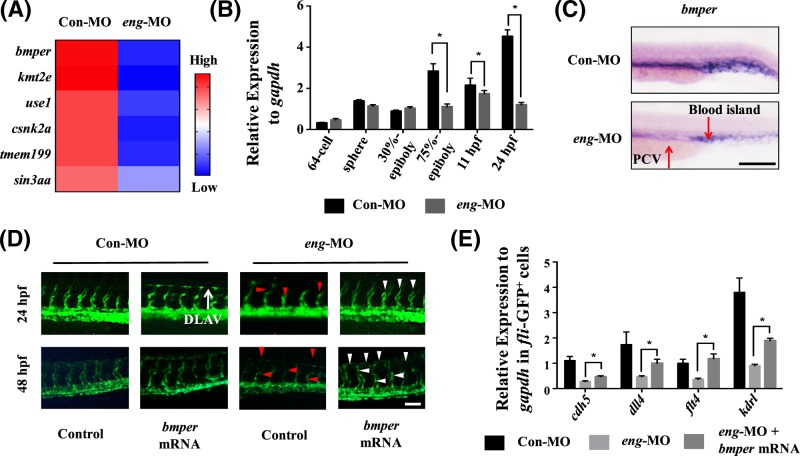
Bmper was involved in endoglin-regulated vasculogenesis (**A**) Heat maps presenting part of the RNA-Seq result. Six vasculogenesis genes were significantly decreased after endoglin knockdown, including *bmper, kmt2e, use1, csnk2a1, tmem199* and *sin3aa.* The level of expression of the gene is indicated with red (high) or blue (low). (**B**) qPCR analysis of bmper expression in different stages of the Con-MO and *eng*-MO group, including 64 cell, sphere, 30% epiboly, 75% epiboly, 11 hpf and 24 hpf stage. *Gapdh* was used as an internal control (mean ± SD; the experiments were repeated three times). (**C**) Bmper expression by WISH in 24 hpf zebrafish embryos of the Con-MO and *eng*-MO group. The red arrow indicates the region where the expression of bmper was significantly decreased. (**D**) Fluorescence image of Con-MO, Con-MO + *bmper* mRNA, *eng*-MO and *eng*-MO + *bmper* mRNA group. (All *Tg(fli1a:EGFP)^y1^* zebrafish embryos were injected with 2 ng morpholinos and 500 pg mRNA. For example, Con-MO group: 2 ng 5-mispair control MO and 500 pg mCherry mRNA.) The red and white arrows point to the disrupted and rescued structure of DLAV and ISVs, respectively. (**E**) qPCR analysis for *cdh5, dll4, flt4* and *kdrl* expression in *fli*-GFP^+^ cells of the Con-MO, *eng*-MO and *eng*-MO + *bmper* mRNA group. *Gapdh* was used as an internal control (mean ± SD; the experiments were repeated three times). A value of *P* was considered statistically significant (^*^P<0.05) for B and E. Scale bars are 100 μm for C and D.

We performed transcriptional profiling by RNA-Seq with 24 hpf embryos of the Con-MO and *eng*-MO groups and then selected six endothelial genes that were exclusively down-regulated, including *bmper, kmt2e, use1, csnk2a1, tmem199* and *sin3aa* (Figure 5A). Previous studies have suggested that endoglin is an associated co-receptor that binds BMP9/10 to activate the Smad in ECs [36]. Bmper, the extracellular BMP modulator, preferentially binds and regulates BMP9 activity in ECs [37]. Moreover, enhancing ALK signal transduction by overexpression of ALK1 and BMP9 could rescue endoglin deficiency (Figure S2A,B). These results suggested that there is a potential link between endoglin and bmper in endothelial ALK signal transduction. Therefore, we tested whether the expression of bmper was regulated by endoglin in ECs. The temporal-spatial expression of bmper in the *eng*-MO group was first checked. The expression of bmper began to decrease after 75% epiboly (Figure 5B) and was significantly reduced in the PCV and caudal vein plexus (CVP) in 24 hpf morphants (Figure 5C). Next, *bmper* mRNA was injected into *Tg(fli1a:EGFP)^y1^* embryos. After bmper overexpression, the 24 hpf Con embryos showed advanced development of the dorsal longitudinal anastomosing vessel (DLAV), and the defective blood vessels of the 24 and 48 hpf morphants were recovered, especially the length of the ISVs (Figure 5D). FACS analysis showed that the ratio of *fli*-GFP^+^ cells was improved to 6.1% in the *eng*-MO + *bmper* mRNA group compared with 3.2% in the *eng*-MO group (Figure S1C,D). For *Tg(flk:GFP)* transgenic zebrafish line, the ratio of *flk*-GFP^+^ cells was improved to 4.5% in the *eng*-MO + *bmper* mRNA group compared with 2.4% in the *eng*-MO group (Figure S1A,B). Moreover, the expression of *kdrl, flt4, cdh5 and dll4* was increased in *fli*-GFP^+^ cells after bmper overexpression in *the eng*-MO group (Figure 5E). These results suggested that bmper could be an important mediator in endoglin-regulated vasculogenesis.

### BMPER increases the expression of ID1 and vessel formation in *ENG* mutant ECs

**Figure 6:**
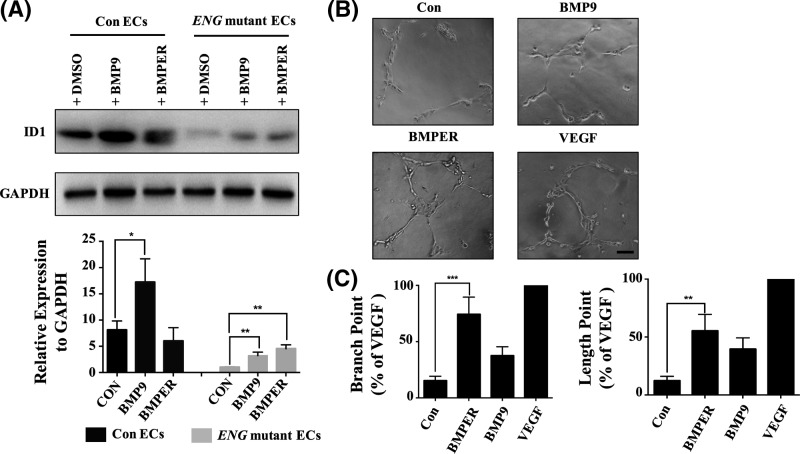
Enhancing the expression of BMPER increased the expression of ID1 and blood vessel formation in *ENG* mutant ECs (**A**) Western blot analysis of ID1 expression in the Con ECs + 0.1% DMSO, Con ECs + 10 ng/ml BMP9, Con ECs + 20 ng/ml BMPER, *ENG* mutant ECs + 0.1% DMSO, *ENG* mutant ECs + 10 ng/ml BMP9 and *ENG* mutant ECs + 20 ng/ml BMPER group. Each group was incubated for more than 24 h at 37°C. GAPDH was used as an internal control. Grey scanning analysis, conducted using ImageJ, was used to analyse the Western blot results (mean ± SD; the experiments were repeated three times). (**B**) The tube formation of *ENG* mutant ECs in four groups, including the Con (EC(FULL)-SFM medium without 30 ng/ml VEGF), VEGF (EC(FULL)-SFM medium), BMP9 (EC(FULL)-SFM medium without 30 ng/ml VEGF + 10 ng/mL BMP9) and BMPER (EC(FULL)-SFM medium without 30 ng/ml VEGF + 20 ng/ml BMPER) group. Tube formation was assessed and photographed at 3 h. Scale bars are 100 μm. (**C**) Quantitative results of tube formation. The number and the length of branches were assessed and counted by Imagine J. The VEGF group was a positive control. The ratio of tube formation = (the number/full-length of branches in each group):(the number/ full-length of branches in VEGF group). Error bars represent the SD of the mean values from three independent experiments. A value of *P* was considered statistically significant (^*^P<0.05, ^**^P<0.01, ^***^P<0.001) for A and C.

To further confirm the functional role of BMPER *in vivo*, we investigated whether human recombinant BMPER (hrBMPER) incubation could rescue endoglin deficiency *in vitro.* Previous reports showed that ECs differentiated from iPSCs derived from patients could be applied to study the molecular pathology of pulmonary arterial hypertension [38]*.* In this study, the transcription factor ID1, a downstream effector of the BMP signalling pathway, was chosen to assess the role of BMPER in BMP signalling transduction in *ENG* mutant ECs. BMP9, a BMP ligand that forms a complex with ALK1 and ENG, was added to activate BMP signalling. Western blotting showed that ID1 decreased in patient *ENG* mutant ECs. After 24 h of hrBMPER treatment, ID1 expression increased approximately 4-fold in *ENG* mutant ECs, which was higher than the expression in BMP9-stimulated cells (Figure 6A). Moreover, BMPER stimulated *ENG* mutant ECs to form more branches than BMP9 in the tube formation experiment (Figure 6B,C). These results suggested that enhancing the expression of BMPER could increase the expression of ID1 and blood vessel formation in *ENG* mutant ECs from HHT patients.

## Discussion

A mutation in the *ENG* gene has been identified in approximately 50% of patients with HHT. Because there is an absence of effective treatments for HHT, identifying the biological functions of endoglin and major downstream effectors in vascular development could provide therapeutic targets for the disease. Hogan and Schulte-Merker highlighted the greater advantages of the zebrafish model in vascular disease research than the mouse model [39]. Therefore, our studies focused on the role of endoglin in ECs and its gene in the zebrafish model. Phylogenetic and sequence similarity studies revealed that *ENG* is a conserved gene among vertebrates. This finding suggests that the endoglin protein in zebrafish could have similar functions as that in humans, providing evidence that zebrafish could be an appropriate experimental model to study endoglin-regulated vascular development *in vivo*.

In zebrafish embryos, endothelial precursors specifically differentiate into endothelial and haematopoietic cells after 75% epiboly [10]. The temporal-spatial profile revealed that the expression of endoglin gradually increases after 75% epiboly in zebrafish embryos and was mainly expressed in the PCV and ISV in the mature vascular system. Baik et al. reported that endoglin integrates BMP and Wnt signalling to regulate early haematopoiesis and vasculogenesis [34]. Therefore, endoglin is believed to be an important receptor that is involved in vascular development.

From the defects in the ISVs of *Tg(fli1a:EGFP)^y^*, we thought that endoglin knockdown might cause abnormal vasculogenesis. Endoglin knockdown in 24 hpf embryos resulted in the decreased expression of EC markers. Considering abnormal endothelium formation leads to AVMs in HHT patients [40], our results suggest that endoglin regulates vascular development by influencing EC differentiation. Interestingly, FACS analysis showed that the ratio of *fli*-GFP^+^ cells was significantly decreased after endoglin knockdown. Because *fli1* is an endothelial and haematopoietic marker in zebrafish [31,32], we also conducted the knockdown experiments on *flk*-GFP^+^ line and got the similar results. FACS results suggest that endoglin could regulated endothelium formation and haematopoiesis in the initial stage of embryogenesis.

Previous studies have reported that endoglin is a TGF β/BMP signalling co-receptor and that the endoglin–BMP9 interaction is essential in ECs [41]. From RNA-Seq data, we selected six vasculogenesis genes whose expression was significantly decreased after endoglin knockdown. Because *bmper* (BMP-binding endothelial regulator) has been reported as a conserved gene that regulates zebrafish haematopoiesis and vascular development [42], we conducted further experiments on its role in endoglin-regulated vascular formation. In our research, the expression of bmper decreased after the 75% epiboly stage in the *eng*-MO group, which represents the stage of haematopoiesis and vasculogenesis. This observation combined with the up-regulated expression of *bmper* during the period of vasculogenesis in parallel to *flk1* expression during mouse embryonic development [27,43] suggests that bmper is highly expressed in the early mesoderm and affects endothelium formation, which is similar to the expression pattern of endoglin in zebrafish. Moreover, Moser et al. provided that bmper knockdown results in disturbed ISVs in zebrafish [42], the phenotype of which is highly similar to that with endoglin knockdown. Our rescue experiments showed that overexpression of bmper could reduce the defects at ISVs after endoglin knockdown and improve the proliferation of ECs. These results confirm that bmper is regulated by endoglin during early development.

In our research, hrBMPER could stimulate *ENG* mutant ECs to form branches and increase the expression of ID1 which is in accordance with the results of *in vivo* zebrafish model, indicating the role of BMPER in promoting BMP signalling and angiogenesis. These results suggest BMPER may rescue endoglin deficiency through its enhancing BMP signalling activity. Although Yao et al. suggested that BMPER inhibits BMP9 [37], it was also reported that BMPER enhances BMP signalling [44,45] and the angiogenic response of ECs in a concentration-dependent manner [27,46]. Moreover, enhancing BMP/ALK signalling by overexpression of *alk1* and *bmp9* could rescue endoglin deficiency.

Previous studies did not fully clarify the molecular pathology of HHT with endoglin mutations, and there is no targeted therapy for those patients. In our research, by combining an in vivo zebrafish model and a HHT patient sample, we first found that BMPER is an important effective protein involved in blood vessel formation regulated by endoglin. In recent years, BMPER has become of increasing interest in EC biology, including its role in cell inflammation [47], atherosclerosis [48] and angiogenesis [27,49–51]. Some research has even analysed the possibility of BMPER as a therapeutic target for several cardiovascular diseases, including pulmonary inflammation and injury [52] and idiopathic pulmonary fibrosis [53]. In this study, BMPER was found to promote blood vessel formation when BMP signalling is deficient in vivo or in vitro, thereby providing a novel strategy to treat BMPER overexpression in HHT. Moreover, BMPER has been reported to play an anti-inflammatory role, which improves the possibility that it can act as a therapeutic target for HHT.

In conclusion, our study suggests that endoglin plays an important role in endothelium formation and vasculogenesis. Bmper is regulated by endoglin and may be a potential therapeutic target for HHT.

## Supporting information

**Supplementary Figure S1:** 

**Supplementary Figure S2:** 

## Abbreviations

Con-MO: injected 5-mispair control morpholino oligonucleotides embryos
CVP: caudal vein plexus
DLAV: dorsal longitudinal anastomosing vessel
EC: endothelial cell
*Eng*: endoglin
*eng*-MO: injected endoglin-morpholino oligonucleotides embryos
HHT: hereditary haemorrhagic telangiectasia
hpf: hours post-fertilisation
hrBMPER: human recombinant BMPER
iPSC: induced pluripotent stem cell
ISV: intersegmental vessel
PCR: polymerase chain reaction
PCV: posterior cardinal vein
PFA: paraformaldehyde
qPCR: quantitative real-time-PCR
RNA-Seq: RNA sequencing
WISH: Whole-mount *in situ* hybridisation
