# Correction: Endoglin is a conserved regulator of vasculogenesis in zebrafish - implications for hereditary haemorrhagic telangiectasia

**DOI:** 10.1042/BSR-2018-2320_COR

**Published:** 2021-10-28

**Keywords:** endoglin, endothelial cells, vasculogenesis, zebrafish

The authors of the original article “Endoglin is a conserved regulator of vasculogenesis in zebrafish – implications for hereditary haemorrhagic telangiectasia” (*Biosci Rep* (2019) **39**(5): BSR20182320; https://doi.org/10.1042/BSR20182320), would like to correct an error in Figure 4C.

In Figure 4C of the published article, the *hey2* and *lmo2 in situ* hybridization images in *eng*-MO are similar. After checking the original data, the authors found that the mistake arose from an alignment error that had occurred whilst they were building their figure on PowerPoint. The correct version of this figure is presented below.

**Figure 4:**
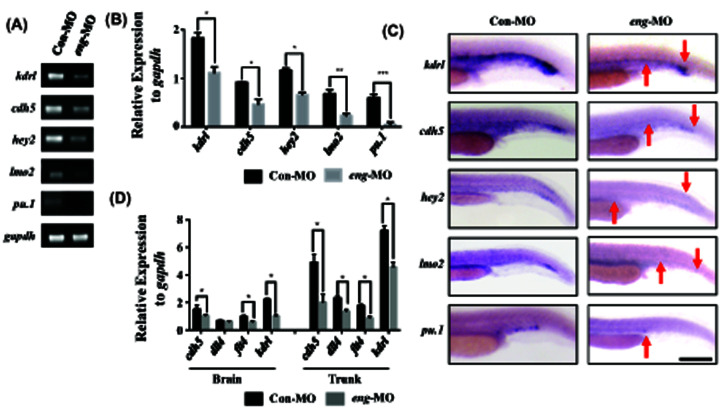
Endoglin knockdown decreased the expression of endothelial markers (**A**) RT-PCR for endothelial markers in the Con-MO and *eng*-MO group, including *kdrl*, *cdh5*, *hey2*, *lmo2* and *pu.1* (A). *Gapdh* was used as an internal control. (**B**) Quantification of mRNA expression in (A) with grey scanning analysis, conducted with ImageJ. (**C**) WISH for endothelial marker expression in the Con-MO and *eng*-MO group among 24 hpf embryos (n>30 for each group). The red arrow indicates the region where the endothelial markers were significantly decreased. Scale bars are 200 μm. (**D**) qPCR for *cdh5*, *dll4*, *flt4* and *kdrl* expression in the brain and trunk of the Con-MO and *eng*-MO group. Gapdh was used as an internal control (error bars, SEM; n=3 biological replicates). A value of P was considered statistically significant (*P<0.05, **P<0.01, ***P<0.001) for B and D.

