# Supplementary material for: Endoglin is a conserved regulator of vasculogenesis in zebrafish – implications for hereditary haemorrhagic telangiectasia
Source: Biosci Rep. 2019 May 21;39(5):BSR20182320. doi: 10.1042/BSR20182320 (PMC6527926; doi:10.1042/BSR20182320)
Supplement: Supplementary file 1 [file bsr20182320_Supp1.pdf]

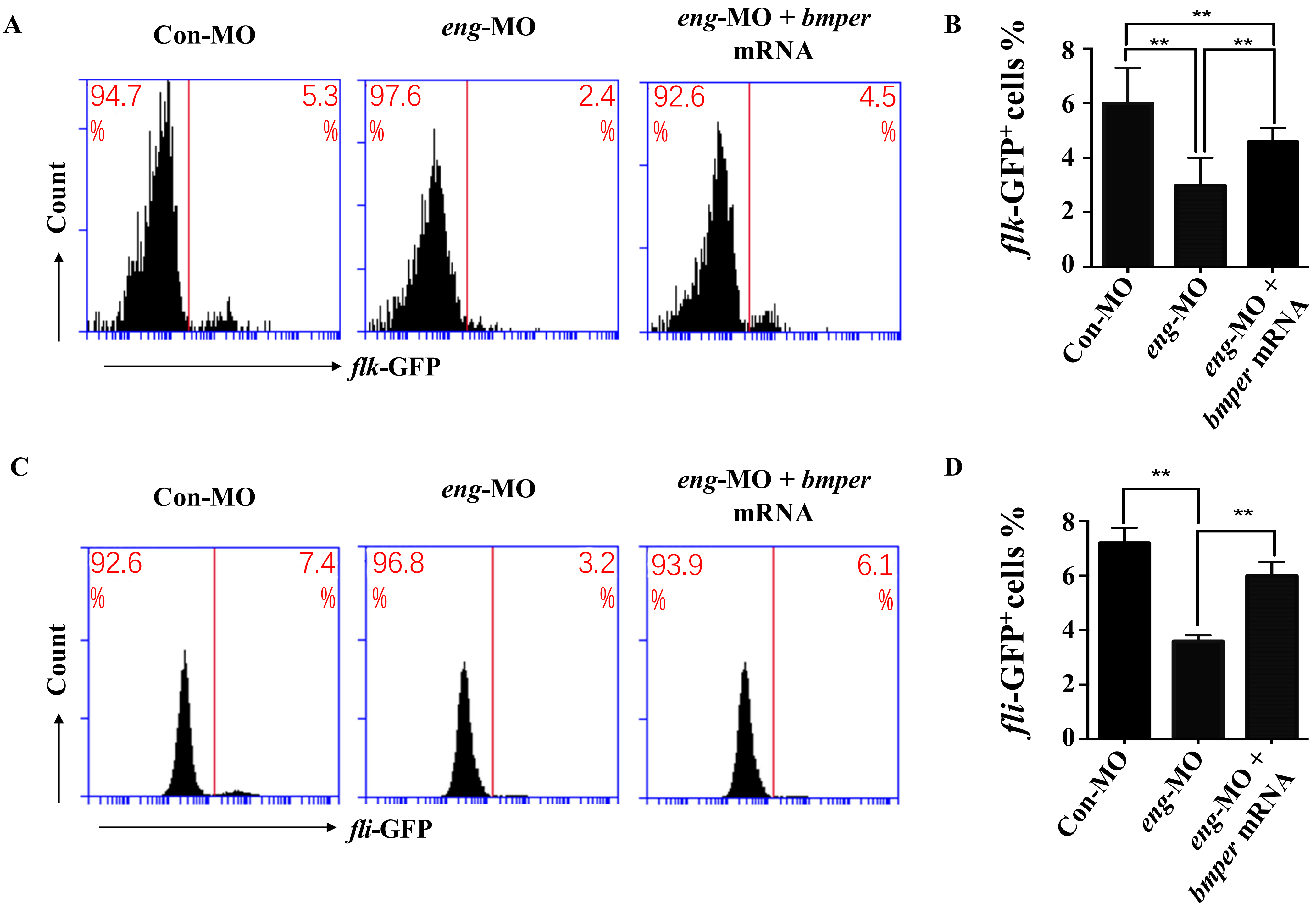

A
Con-MO
eng-MO
eng-MO + bmper mRNA
94.7 %
5.3 %
97.6 %
2.4 %
92.6 %
4.5 %
Count
flk-GFP
B
flk-GFP+ cells %
0
2
4
6
8
**
**
**
Con-MO
eng-MO
eng-MO + bmper mRNA
C
Con-MO
eng-MO
eng-MO + bmper mRNA
92.6 %
7.4 %
96.8 %
3.2 %
93.9 %
6.1 %
Count
fli-GFP
D
fli-GFP+ cells %
0
2
4
6
8
**
**
Con-MO
eng-MO
eng-MO + bmper mRNA

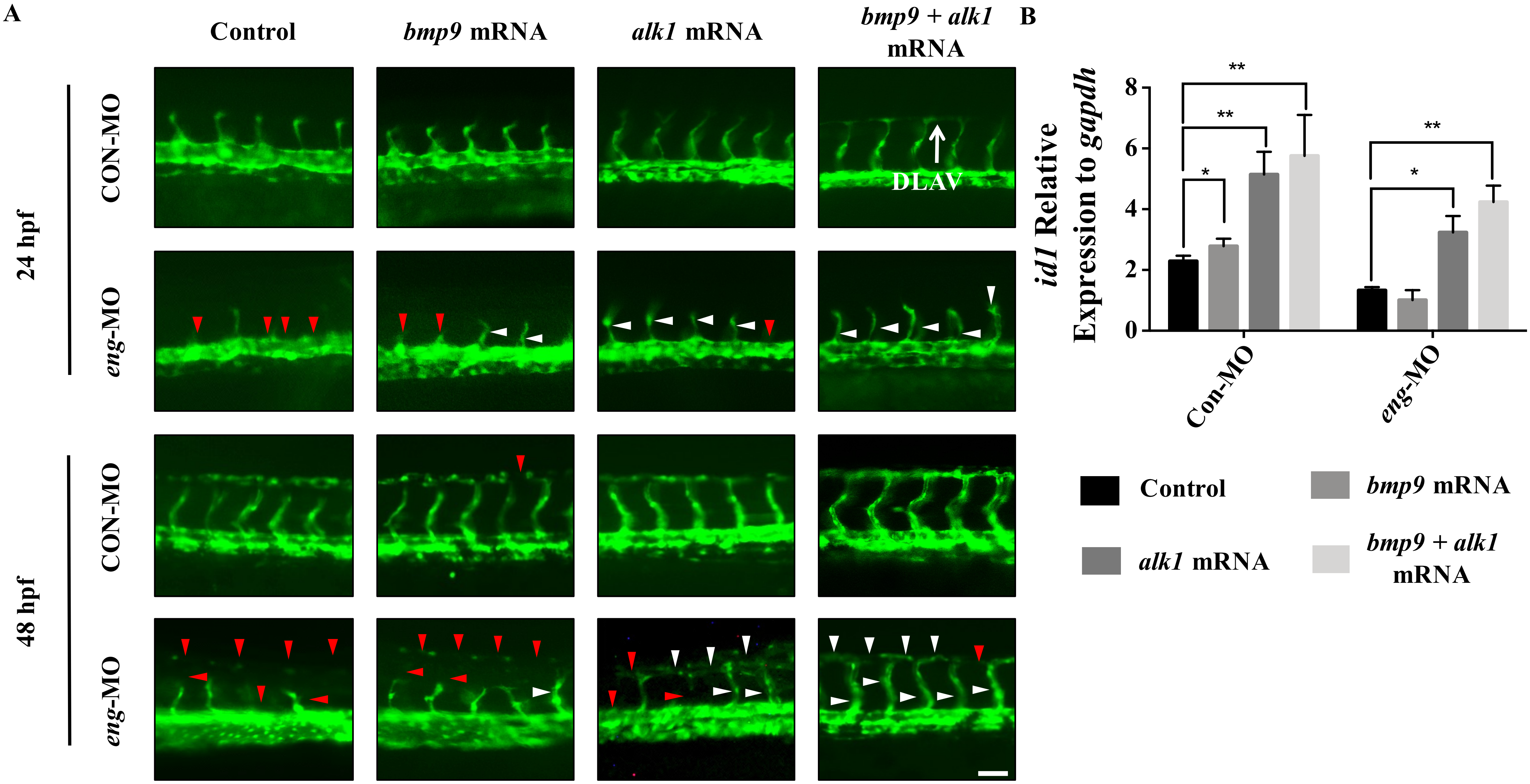
A
Control
bmp9 mRNA
alk1 mRNA
bmp9 + alk1 mRNA
24 hpf
48 hpf
CON-MO
eng-MO
CON-MO
eng-MO
DLAV
B
id1 Relative Expression to gapdh
8
6
4
2
0
**
**
*
**
*
Con-MO
eng-MO
Control
bmp9 mRNA
alk1 mRNA
bmp9 + alk1 mRNA
